# Spinal cord injury due to fall from electricity poles after electrocution

**DOI:** 10.12669/pjms.35.4.241

**Published:** 2019

**Authors:** Amir Zeb, Aatik Arsh, Sher Bahadur, Syed Muhammad Ilyas

**Affiliations:** 1 Amir Zeb, PPDPT, MSPT, Senior Physical Therapist, Paraplegic Center, Peshawar, Pakistan; 2 Aatik Arsh, DPT, MSPT, Physical Therapist, Paraplegic Center, Peshawar, Pakistan; 3 Sher Bahadur, MSc. HPM (Health Policy and Management), Senior Research Officer, Rehman Medical College, Peshawar, Pakistan; 4 Syed Muhammad Ilyas, MSPT, Chief Executive Officer, Paraplegic Center, Peshawar, Pakistan

**Keywords:** Fall, Electric injuries, Epidemiology, Spinal cord, Rehabilitation

## Abstract

**Objective:**

The objective of this study was to report epidemiology, complications and rehabilitation outcomes of patients who sustained spinal cord injury (SCI) due to fall from electricity poles after electrocution.

**Methods:**

A prospective observational study was conducted in which patients admitted to Paraplegic Centre Peshawar from July 2016 to July 2018 who sustained SCI due to fall from electricity poles after electrocution were included. Of total 852 patients, 39 (4.58%) sustained SCI due to fall from electricity poles after electrocution. Two patients were excluded and data of 37 patients was analyzed.

**Results:**

The mean age of the participants was 35.03±13.47 years. Twenty-two (59.4%) patients had associated burns on different parts of body. Twenty-seven (72.9%) had pressure ulcers, 31 (83.8%) had spasticity, 18 (48.6%) had neuropathic pain and 2 (5.4%) had limb amputations due to injury. Mean Spinal cord injury independence measure score at the time of discharge was 53.4±5.7.

**Conclusion:**

SCI due to fall from electricity poles after electrocution is rare however combined effect of injury by electricity along with fall from electricity poles are associated with severe complications. Rehabilitation outcomes in these patients are also minimal

## INTRODUCTION

Spinal cord injury (SCI) is a devastating condition and is associated with incredible costs and human sufferings.^1^ It is reported that annual incidence of SCI in developed countries is between 10 to 80 cases per million annually.^2^ However, data from developing countries is scarce. Road traffic accidents, weight fallen over, fire arm injuries and fall from trees, roofs etc. are the leading causes of traumatic SCI.^3^ Beside these, other minor causes include bomb blast injuries, diving accidents and stab injuries.^4^ Fall from electricity poles after electrocution is also an uncommon cause of SCI which is rarely described in literature.^5^

High-voltage electrical current lead to major injuries to organ system in the body.^5^ Integumentary and neurological system is commonly injured in electrical injuries.^6^ Due to limited studies regarding SCI sustained due to electric shock related fall, that’s why its exact prevalence is not reported. Gaur R at al. reported 1.91% prevalnce of electric shock related SCI.^7^ Electric shock related neurological injuries have unique pattern and is associated with more complications as compared to other injuries.^8^

Literature search revealed that quite a few studies have reported epidemiology, clinical characteristics and complications of spinal cord injury patients in Pakistan.^9^ However none of these studies highlighted SCI due to fall from electricity poles after electrocution. Therefore, there was a dire need to conduct this study in order to determine epidemiology, clinical characteristics, complications and rehabilitation outcomes of patients who sustained SCI due to fall from electricity poles after electrocution.

## METHODS

A prospective observational study was conducted at Paraplegic Centre Peshawar. Patients admitted to Paraplegic Centre Peshawar from July 2016 to July 2018 who sustained SCI due to fall from electricity poles after electrocution were included in the study. Those patients were excluded who either died or leave against medical advice (LAMA).

Ethical approval was obtained from Ethics committee of Paraplegic Centre Peshawar. Informed consent was obtained from all those patients who admitted to Paraplegic Centre Peshawar with history of SCI due to fall from electricity poles after electrocution. Out of total 852 participants admitted in the study period (July 2016 to July 2018), only 39 (4.58%) sustained SCI due to fall from electricity poles after electrocution. A thorough clinical and radiological examination of all these 39 patients was performed, however one patient died during inpatient rehabilitation because of chest complications and one patient left against medical advice (LAMA), that’s why data of only 37 patients were analyzed.

Information regarding demographics, physiological intactness of spinal cord injury, neurological level, presence or absence of complications and co-morbidities at the time of admission to rehabilitation center was noted. The American Spinal Injury Association (ASIA) classification system was used to classify injuries as complete (ASIA A) or incomplete (ASIA B, C, D or E). Modified Ashworth scale was used to report spasticity in lower limbs while Leeds Assessment of Neuropathic Symptoms and Signs Pain Scale (English version) was used to differentiate neuropathic pain from nociceptive and visceral pain. Spinal cord injury independence measure (SCIM) was used at the time of discharge to report rehabilitation outcomes. Trained physical therapist with master in physical therapy degree and three years’ experience in spinal cord injury rehabilitation center collected the data from the participants. All data were initially documented on pages and then entered into SPSS sheet. In order to ensure confidentiality, all the data was carefully coded. Moreover, the data was carefully stored in password locked computer and access to the data was available only for selected people who were actively involved in the data collection. SPSS version 23 was used to analyze the data.

## RESULTS

The mean age of the participants was 35.03±13.47 years and all 37(100%) participants were male. Twenty three (62.2%) participants were from Khyber Pakhtunkhwa, 10 (27.0%) from tribal area and remaining 4(10.8%) were from other provinces of Pakistan. Two third of participants (n=28, 75.7%) were married, while 9(24.3%) were single at the time of injury. Eleven (29.7%) participants were uneducated while remaining 26(70.3%) were having different levels of education (From primary level to Master level). By profession, 10 (27.0%) participants were professional electricians/ WAPDA employees, 10(27.0%) were laborer, 6(16.2%) were farmers, 3 (8.1%) were teachers, 2 (5.4%) were students and remaining 6 (16.2%) were having no specific profession.

Majority 26(70.2%) of the patients had complete thoracic paraplegia, 3 (8.10%) had complete and 3 (8.10%) had incomplete cervical tetraplegia, 3 (8.10%) had Complete lumbar paraplegia while 1(2.70%) had Incomplete thoracic and 1(2.70%) had Incomplete lumbar paraplegia. Sixteen (43.2%) patients underwent spine fixation surgery while 21 (56.8%) patients were managed conservatively. Twenty two (59.4%) patients had associated burns on different parts of body (Table-I).

**Table I:** Complications/Co-morbidity among patients who sustained spinal cord injury due to fall from electricity poles after electrocution.

Complication/ Co-morbidity	Frequency/ Percentages	Sub classification of Complication/Co-morbidity	Frequency/ Percentages
Burns	22(59.4%)	Head and Neck	2(5.4%)
		Chest	2(5.4%)
		Upper limbs	
		Upper arm	1(2.7%)
		Forearm	1(2.7%)
		Hands	7(18.9%)
		Fingers	4(10.8%)
		Lower limbs	
		Knee	3(8.1%)
		Feet	2(5.4%)
Pressure ulcer	27 (72.9%)	Single PU	11 (29.7%)
		Multiple PU	16 (43.2%)
Spasticity	31 (83.8%)	1	13 (35.1%)
		1+	7(18.9%)
		2	6 (19.3%)
		3	4(10.8%)
		4	1 (2.7%)
Deep venous thrombosis	3 (8.1%)	Rt. Lower limb	1 (2.7%)
		Lt. Lower limb	2(5.4%)
Neuropathic pain	18 (48.6%)	At level neuropathic pain	7 (18.9%)
		Below level neuropathic pain	11 (29.7%)
Hepatitis	2 (5.4%)	Hepatitis B	1 (2.7%)
		Hepatitis C	1 (2.7%)
Amputation	2 (5.4%)	Rt. Below elbow	1 (2.7%)
		Lt. Above knee	1 (2.7%)

Mean SCIM score at the time of discharge was 53.4±5.7. Analysis of subsections of SCIM showed that mean self-care score was10.6±0.9, mean respiration and sphincter management score was 24.5±2.5 while mean mobility score was 18.3±1.7.

## DISCUSSION

Electric shock related spinal cord injury is uncommon, that’s why it didn’t receive much attention. Gaur R et al.^7^ reported 1.91% while Lammertse DP reported less than 0.1% prevalnce of electric shock related SCI.^10^ However results of current study has shown that 4.58% patients sustained SCI due to fall from electricity poles after electrocution. This shows that electric related injuries are more common in Pakistan as compared to other countries. As there were no previous studies available which reported electrical related SCI in Pakistan that’s why it was not possible to compare results of current study with previous studies from Pakistan however there are reports that electrical injuries are high in Pakistan due to poor compliance with safety measures.^11^,^12^ The alarming fact highlighted by current study was that only 27.0% participants were professional electricians/WAPDA employees while the remaining all participants were having other profession but still they were working on electricity poles.

In most cases electrical injuries leads to devastating results. Though neurological injuries are not commonly reported in minor electrical injuries however they are common in major electrical injuries.^6^,^13^ Neurological injuries due to electricity can be due to direct result of electricity because of thermal injury, electroporation, and vascular damage and can be due to indirect cause because of fall or hitting an object. No matter, what is the reason, neurological involvement in electrical injuries are associated with hard to believe consequences.^10^

Majority of participants in current study had complete thoracic paraplegia. Similar results are also reported by other studies conducted in Pakistan.^14^-^17^ Burns were common among participants at initial presentation. Electricity related injuries are almost always associated with burns.^6^ The most common site of burn was upper limb. This may be due to the fact that previous studies reported that entry site in majority of electrical injury cases are hand.^7^ Pressure ulcers, spasticity and neuropathic pain were common secondary complications among these participants. Literature also reported high prevalence of these secondary complications among spinal cord injury patients.^3^,^14^,^18^ Injury related amputations among SCI patients is uncommon and majority of literature available regarding it, is available is in the form of case reports.^19^,^20^ Two cases, one with below elbow and one with above knee amputation were included in current study.

Though SCI is incurable but rehabilitation strategies aim to minimize complications and maximize independence according to patient functional capabilities^18^,^21^-^23^ All patients included in current study underwent comprehensive physical rehabilitation protocols at Paraplegic Centre Peshawar. No study is found in literature which reported rehabilitation outcome of patients who sustained SCI due to fall from electricity poles after electrocution however SCIM scores of participants in current study showed that majority of patients who sustained SCI due to fall from electricity poles after electrocution did not achieve maximal independence as compared to their counterparts with SCI due to other causes.

### Limitations of the study

Despite the fact that current study was first of its kind which reported epidemiology, clinical characteristics, complications and rehabilitation outcomes of patients who sustained SCI due to fall from electricity poles after electrocution, yet it has some limitations. First of all, current study was conducted in clinical settings due to which it was difficult to control confounding variables. Second, it was difficult to isolate whether SCI occured due to direct electrical injury or due to fall. Moreover, all these patients were initially managed in different tertiary care hospitals, each with its own protocols for managing SCI patients. This definitely affected overall outcomes, but difficult to trace as they were beyond the scope of current study.

### Suggestions

There is a dire need to develop clinical guidelines for the management of SCI patients who sustained injury due to fall from electricity poles after electrocution. Moreover, it is recommended to conduct large multicenter clinical trials in order to truly determine rehabilitation needs of these patients.

## CONCLUSION

SCI due to fall from electricity poles after electrocution is rare however combined effect of injury by electricity along with fall from electricity poles are associated with severe complications. Burns and amputation are unique complications associated with SCI due to fall from electricity poles after electrocution. Rehabilitation outcomes in these patients are also minimal as compared to their counterparts with SCI due to other causes.

### Author`s Contribution

**AZ:** Acquisition of data, drafting the manuscript.

**AA:** Concept and study design, analysis & interpretation of data, drafting the manuscript, Critical revision.

**SB:** Literature search and literature review, Critical revision.

**SM:** Analysis & interpretation of data, Critical revision, final approval of the version to be published.
